# Spatial differentiation of productive services and its influencing factors: A case study of Kunming, China

**DOI:** 10.1371/journal.pone.0326845

**Published:** 2025-07-24

**Authors:** Tao Yang, Li Ma

**Affiliations:** Creation Research and Development Institute, Yunnan Design Institute Group Engineering Investment Co., Ltd, Kunming, China; Yunnan University, CHINA

## Abstract

The transition to a service-based economy represents a key global macroeconomic trend, with productive services playing a critical role in driving economic growth. For China, the development of productive services is a strategic priority in its pursuit of high-quality development. Most existing research primarily relies on traditional data to examine the spatial agglomeration and influencing factors of productive services in economically advanced regions, often overlooking the integration of multi-source data and spatial analyses in less developed areas. This study focuses on Kunming as the case study, employing methods such as Standard Deviation Ellipses (SDE), Kernel Density Estimation (KDE), and Local Spatial Autocorrelation (Moran’s I) to investigate its spatial differentiation and agglomeration patterns. Additionally, Geodetector is applied to analyze influencing factors, utilizing multi-source data including Point of Interest(POI), LandScan, the annual China Land Cover Dataset (CLCD), OpenStreetMap (OSM), and socio-economic data to examine the evolutionary patterns of productive services. The findings suggest that Kunming’s productive service sectors currently exhibit a predominant southward diffusion, influenced primarily by transportation infrastructure and economic conditions. Moreover, different categories of productive services exhibit unique spatial differentiation and influencing factors. Moving forward, it is essential to prioritize upgrading the internal structure of productive services to foster sustainable and high-quality sectoral development.

## 1. Introduction

Against the backdrop of a global shift towards a service economy in macroeconomic development, productive services are playing an increasingly important role. Productive services are highly specialized, innovative, and have strong industry integration and radiation capabilities; they are the strategic high ground of global industrial competition and the core engine driving industrial transformation, upgrading, and changes in development modes [1]. In the context of China’s industrialization and urbanization, accelerating the development of productive services not only enhances the level of industrialization but also facilitates the optimization and upgrading of the industrial structure—representing a “win-win” strategic approach to achieving high-quality economic development [2,3]. As digital transformation accelerates, productive services have increasingly become integral in driving technological innovation within the manufacturing sector. Their contributions to industrial productivity, knowledge accumulation, and the market environment further advance China’s innovation-driven development strategy.

The spatial agglomeration of productive services is significantly influenced by regional economic disparities and the optimization of industrial spatial layouts [4,5]. In relation to regional economic disparities, the concentration of productive services is recognized as a key factor in driving economic growth, particularly in fields such as scientific research and finance [6]. A strong correlation has been observed between urban productivity and the agglomeration of productive services, though its impact varies across regions [7]. Furthermore, while specialized agglomeration may have negative effects on environmental quality, diversified agglomeration is found to improve it, suggesting that a balance between different types of agglomeration is necessary when formulating development strategies [8]. In terms of optimizing industrial spatial layout, the synergetic clustering of productive services and manufacturing is found to enhance regional innovation efficiency, although its impact also varies by region and industry type [9]. Significant changes in infrastructure, such as the development of high-speed rail networks, have altered the spatial layout of productive services, further promoting specialized clustering [10]. Overall, while the spatial concentration of productive services drives regional economic growth and innovation, it also brings challenges such as environmental impacts and regional disparities. Therefore, policy-making must prioritize balancing aggregation and diffusion processes to optimize industrial spatial layouts, ultimately aiming for the achievement of sustainable development goals.

The study on the spatial differentiation of productive service industries reveals significant characteristics of clustering in urban core areas and diffusion towards the periphery, driven by multiple dynamic mechanisms [2,11,12]. These patterns and their mechanisms show significant differences across different regions in China (eastern, central, and western). In terms of spatial differentiation and clustering features, productive service industries are found to cluster in urban core areas, thereby enhancing local productivity and innovation capabilities. This clustering is generally accompanied by a spatial spillover effect that positively impacts surrounding areas. However, this spillover effect is more pronounced in eastern regions while it remains relatively limited in central and western regions [13,14]. Regional differences further indicate that in the eastern region, the agglomeration of productive services has significantly promoted improvements in urban productivity and environmental quality, while in the central and western regions, its effects depend on specific local conditions [11]. Regarding driving mechanisms, knowledge spillover, locational factors, urban hierarchy structure, and government policies are identified as crucial factors influencing spatial differentiation in productive services. Foreign direct investment (FDI) and industrial agglomeration have been shown to significantly enhance knowledge spillover, especially in cities with specialized industrial structures and high absorption capacity [15]. Locational factors, including urbanization level, land use, and economic conditions, play a crucial role in shaping the spatial distribution of productive services; eastern regions rely more on the expansion of urban construction land while urbanization rates are more decisive in central regions [16]. Moreover, the urban hierarchy structure (such as core cities like Chengdu and Chongqing) plays a key role in economic connections and spatial patterns; meanwhile, government policies promoting industrial synergetic clustering have had differing impacts on regional development [17]. In terms of regional focus, the spatial spillover effect of the productive service industry in the western region is relatively weak, with the enhancement of its ecological efficiency primarily dependent on local agglomeration rather than regional spillover [18]. In contrast, the diverse agglomeration patterns in the central and eastern regions substantially contribute to productivity and environmental quality improvements.

As an important city in China’s western region, Kunming’s productive service industry plays a key role in the transformation of the regional economy. As the core of the central Yunnan urban agglomeration, Kunming has a significant radiating and driving effect on regional economic development, with an important contribution to promoting regional integration and urban expansion [19]. However, compared to the central and eastern regions, Kunming’s productive services show significant differences in agglomeration patterns and transformation needs. The agglomeration effect of Kunming’s productive services is relatively weak, lacking notable spatial spillover effects, while the eastern region has a more pronounced agglomeration effect [13]. Additionally, Kunming’s development relies more on MAR externalities brought by similar industrial clusters rather than the diversified agglomeration models of the eastern region. This indicates that Kunming needs to formulate localized development strategies that focus on enhancing agglomeration benefits and promoting deep integration between productive services and manufacturing for economic growth and environmental sustainability [20]. Therefore, studying Kunming’s productive services not only has theoretical value but also provides a practical reference for economic development in western regions.

In the study of productive service industries, commonly employed methods include spatial econometric models, GIS-based spatial analysis, and spatial autocorrelation, all of which focus on quantifying spatial differentiation and uncovering the spatial patterns of the services sector. For example, location quotient and spatial Gini coefficient are traditional quantitative methods that can effectively measure concentration and distribution characteristics of productive service industries to help identify areas with above or below-average service levels and reveal their spatial differentiation [21]. Meanwhile, GIS is widely used for visualization analysis of spatial data. Combined with statistical methods such as spatial autocorrelation and kernel density analysis, it can more intuitively present spatial patterns and relationships. The combination of GIS with quantitative analysis is mainly reflected in techniques like spatial clustering analysis, research on spatial autocorrelation, and pattern abstraction; using tools like ArcGIS allows researchers to analyze the characteristics of space distribution and agglomeration in the services industry more clearly [21–23]. In regions such as Kunming, research using GIS has analyzed the spatial distribution patterns of everyday consumer venues, revealing characteristics of spatial aggregation and heterogeneity. However, the application of GIS and quantitative methods in underdeveloped areas is still relatively limited. Further localized studies are needed in regions like Kunming to reveal their unique spatial dynamics. Additionally, there is a lack of research integrating socio-economic data with spatial data; such integration helps to more comprehensively analyze how socio-economic variables influence the spatial differentiation of productive services.

This study focuses on the productive service industry in Kunming as the research subject. Utilizing Point of Interest(POI) data, it examines its spatial differentiation and agglomeration characteristics through standard deviation ellipses(SDE), kernel density estimation(KDE), and local spatial autocorrelation analysis. Furthermore, by incorporating POI, LandScan, the annual China Land Cover Dataset (CLCD), OpenStreetMap (OSM), and socio-economic data, the study explores the influencing factors through geographic detectors to analyze the evolutionary patterns. Based on these analyses, recommendations for spatial optimization and future development of the productive service industry are proposed. The study provides a more comprehensive analysis of the spatial differentiation and influencing factors of the productive service industry, while further investigating its spatiotemporal evolution patterns. The findings offer a foundation for evaluating the rationality of spatial layouts in the productive service sector, facilitating structural adjustments and optimization upgrades in Kunming’s service industry, and contributing to high-quality regional economic development.

## 2. Materials and methods

### 2.1. Research area

**Fig 1:**
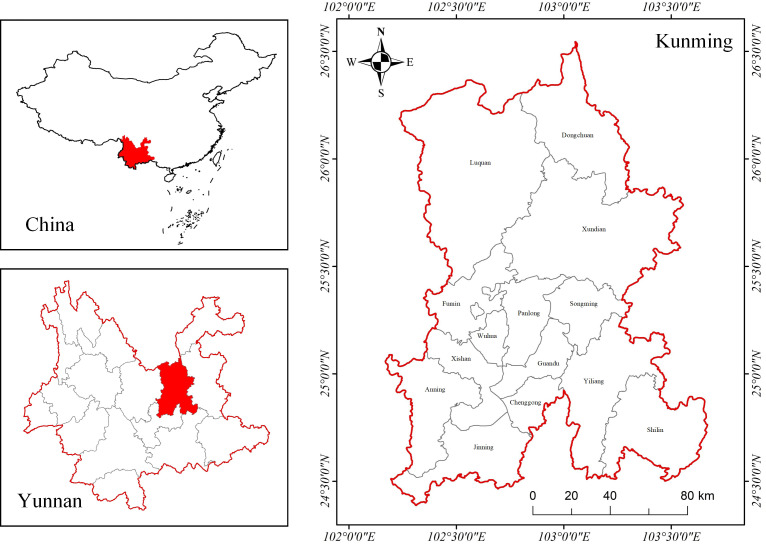
Study area. Note: Republished from [http://bzdt.ch.mnr.gov.cn/] under a CC BY license, with permission from [the Ministry of Natural Resources of the People’s Republic of China], original copyright [2022].

Kunming is situated in the central part of the Yunnan-Guizhou Plateau, at the intersection of the China-ASEAN Free Trade Area, the Lancang-Mekong Cooperation region, and the Pan-Pearl River Delta economic circle. To the east, Kunming connects Guizhou and Guangxi provinces to the southeast coast of China. To the north, Kunming passes through Sichuan Province and Chongqing to connect with the Central Plains. To the south, Kunming passes through Vietnam and Laos to Southeast Asian countries. To the west, Kunming passes through Myanmar on its way to South Asian countries. It can be seen that Kunming has a unique location advantage and is the forefront of Yunnan’s construction of the “One Belt, One Road” and the “central processor” of the urban economic circle in Central Yunnan (Fig 1). In this context, Kunming’s industrial structure is undergoing a gradual transformation and upgrading. By 2023, the service sector contributed 66.5% to the GDP. While the development trend of productive services in Kunming has been positive in recent years, challenges such as an underdeveloped industrial structure and imbalanced development persist, necessitating further optimization and adjustment.

### 2.2. Data source

#### 2.2.1. POI.

The POI data used in this study is related to producer services in Kunming in 2022. After spatial matching and deduplication, 58444 POI data were obtained, which were then classified according to service types (Table 1) [24]. This study employs POI data to examine the spatial distribution characteristics of productive services in Kunming.

**Table 1:** POI industry breakdown statistics of productive services.

Classification	POI categories	Data summation	proportion
financial productive services	Financial institutions, insurance companies, banks, securities companies, ATMs, etc	18131	31.02%
science and education productive services	Scientific research institutions, training institutions, scientific and educational cultural venues, media organizations, driving schools, etc	15983	27.35%
other productive services	Logistics, post office, intermediary agency, power and telecommunications business hall, maintenance site, talent market, etc	24330	41.63%
total	——	58444	100.00%

Unlike the consumer service industry, the service target of productive services is the producer. According to the Statistical Classification of Productive Services (2019) by the National Bureau of Statistics, the statistical framework for productive services is divided into 10 categories, 35 subcategories, and 171 detailed subcategories. In this study, 16 middle classes that are highly dependent on urban internal spatial elements are screened and integrated into 3 types of productive services, namely financial productive services(FPS), science and education productive services(SEPS), and other productive services(OPS).

#### 2.2.2. CLCD.

The annual China Land Cover Dataset (CLCD), classified by the Institute of Remote Sensing Information Processing at Wuhan University, is derived from Landsat imagery processed via Google Earth Engine. The classification results exhibit strong concordance with global datasets on forest dynamics, surface water, and impervious surfaces [25]. The dataset encompasses nine distinct land cover categories, including impervious surfaces, cropland, forests, water bodies, and shrublands. In this research, the CLCD was employed to delineate the built-up regions of Kunming.

#### 2.2.3. LandScan.

The population data utilized in this study were sourced from the LandScan database, developed by Oak Ridge National Laboratory. The dataset employs the most reliable available census data to construct a weighted model. With a spatial resolution of 1 km, the database offers global population distribution data at a higher resolution, thus providing a more accurate depiction of spatial population distribution [26,27]. In this research, the 2021 LandScan data for Kunming are applied to analyze urban population density.

#### 2.2.4. OSM.

Road network data for this study were sourced from the OpenStreetMap (OSM) platform. OpenStreetMap is an online crowdsourced mapping service that provides access to a wide range of geospatial data. OSM aims to offer free and accessible digital map resources to users. As the most widely utilized platform for Volunteered Geographic Information (VGI), it includes fundamental spatial information and attribute data [28], and is renowned for its high accuracy in road data and the quality of its topological relationships. In this study, OSM data for Kunming from 2021 were employed to assess the traffic conditions within the urban agglomeration by calculating road network density.

#### 2.2.5. DEM.

The Digital Elevation Model (DEM) is an essential tool for terrain analysis, including the assessment of slope, aspect, and hydrological features. In this study, DEM data were sourced from the Geospatial Data Cloud platform. These data were utilized to examine the spatial differentiation of the service industry in relation to terrain factors, as discussed in this paper.

#### 2.2.6. GDP spatial distribution grid data.

The data represents the spatial distribution of China’s GDP in 2019 at a kilometer grid level, sourced from the Resources and Environmental Science and Data Center of the Institute of Geographic Sciences and Natural Resources Research, Chinese Academy of Sciences [29]. This data is generated through spatial interpolation of 1km×1km spatial grid data. In this study, economic factors influencing the spatial differentiation of urban productive services are analyzed using the GDP spatial distribution grid data for Kunming.

In summary, all types of research data provide relatively comprehensive information, facilitating a thorough analysis of the factors influencing the spatial differentiation of productive services in Kunming. The various data sets will be processed using ArcGIS with a 1km×1km grid to standardize their resolution. The relevant information is presented in Table 2. Although the third-party datasets used in this study provide rich information and are helpful for analyzing the spatial differentiation characteristics of the productive service industry in Kunming, the potential biases of the above-mentioned datasets may affect the accuracy of the research results. Therefore, when interpreting the research results, the possible impact of these data biases should be considered, and measures should be taken in subsequent studies, such as using multi-source data validation and improving data quality control, to enhance the reliability of the research results.

**Table 2:** Basic information of various research data.

Type	Source	Resolution	Release time
POI	www.amap.com	——	2014-2022
DEM	https://www.gscloud.cn/search	30m × 30m	2022
GDP	https://www.resdc.cn/DOI/DOI.aspx?DOIID=33	——	2022
CLCD	https://zenodo.org/records/8176941	30m × 30m	2022
LandScan	https://landscan.ornl.gov/	1000m × 1000m	2022
OSM	http://lwww.openstreetmap.org	——	2022

### 2.3. Research methods

#### 2.3.1. Standard deviational ellipse.

In this study, Standard Deviation Ellipse (SDE) analysis is applied to describe the spatial distribution pattern and macro-level changes of productive services in Kunming. The choice of SDE was primarily driven by its ability to effectively summarize key spatial characteristics, including central tendency, dispersion, and directional trends of geographic phenomena. SDE has been widely used in studies of spatial patterns of geographic features due to its straightforward and intuitive representation of spatial data. Specifically, it allows for clear visualization of the spatial extent and orientation of clusters of points, such as productive services in urban areas [30].

The central tendency of each element is represented by the mean central coordinate of the SDE. The dispersion of the element is reflected by the area of the ellipse and its minor axis. The primary directional trend of each element is indicated by the azimuth angle. The greater the difference between the major and minor axes, the more pronounced the directional tendency of the elements. Depending on the standard deviation of the input elements, the ellipse will encompass a varying proportion of the elements: one standard deviation encompasses approximately 68% of the total, two standard deviations cover about 95%, and three standard deviations encompass roughly 99% of the total elements’ centers of mass. Based on the KDE of POI data, this study further analyzes the spatial differentiation of productive services in Kunming using SDE with two standard deviations.

Assume that the coordinates of all points in the productive services point data set are $\left(x_{1},y_{1}\right),\left(x_{2},y_{2}\right),\cdots \left(x_{n},y_{n}\right)$, and tanθ as the direction of the standard variance ellipse, the maximum standard deviation distance $\sigma_{x}$ is the length of the major axis of the ellipse, and the minimum distance $\sigma_{y}$ is the length of the minor axis of the ellipse. The direction Angle, long axis length and short axis length of SDE are calculated as follows:


$$\tan\theta =\frac{\sum_{i}^{n}=1{\tilde{x}}_{i}^{2}-\sum_{i}^{n}=1{\tilde{y}}_{i}^{2}+\sqrt{{\left(\sum_{i}^{n}=1{\tilde{x}}_{i}^{2}-\sum_{i}^{n}=1{\tilde{y}}_{i}^{2}\right)}^{2}+4{\left(\sum_{i}^{n}=1{\tilde{x}}_{i}{\tilde{y}}_{i}\right)}^{2}}}{2\sum_{i}^{n}=1{\tilde{x}}_{i}{\tilde{y}}_{i}}$$ (1)

$$\sigma_{x}=\sqrt{\frac{\sum_{i=1}^{n}{\left[\left(x_{i}-x\right)cos-\left(y_{i}-y\right)\sin\right]}^{2}}{n}}$$ (2)

$$\sigma_{y}=\sqrt{\frac{\sum_{i=1}^{n}{\left[\left(x_{i}-x\right)sin-\left(y_{i}-y\right)\cos\right]}^{2}}{n}}$$ (3)

Where ${\tilde{x}}_{i}$ and ${\tilde{y}}_{i}$ are the difference between the central coordinates of the mean (x, y) and the characteristic coordinates ($x_{i}$, $y_{i}$), respectively. θ is the rotation direction Angle.

#### 2.3.2. Kernel Density Estimation.

Kernel Density Estimation (KDE) is a non-parametric method used to estimate the density of elements around each point, revealing spatial patterns without assuming any specific data distribution. It is particularly useful when the spatial data distribution is unknown or irregular, as it avoids the constraints of parametric models.

In this study, KDE was used to identify the spatial structure of productive services in Kunming. The method provides a clear visualization of service concentration and spatial clustering, making it ideal for understanding how productive services are distributed across the city. The flexibility of KDE allows it to capture complex, non-uniform distributions of services, which is essential for analyzing the spatial differentiation of productive services.

Its calculation formula is as follows:


$$f(s)=\sum_{i=1}^{n}\frac{1}{h^{2}}k(\frac{s-c_{i}}{h})$$ (4)

Where: f(s) is the estimated nuclear density at element s n is the number of elements whose distance from s is less than or equal to h h is the search radius; k is the spatial distance weight.

Additionally, different search radii can yield varying results. Therefore, the outcomes of KDE are influenced by the validated search radius. The formula for determining the search radius is as follows:


$$0.9\times \min\left(SD,D_{m}\times \sqrt{\frac{1}{\ln2}}\right)\times n^{-0.2}$$ (5)

Where SD represents the standard distance, $D_{m}$ represents the median distance, and n represents the number of event points.

#### 2.3.3. Anselin Local Moran’s I.

The first law of geography states that spatial features exhibit autocorrelation, meaning nearby locations influence each other. In this study, Anselin’s Local Moran’s I statistic was used to analyze the spatial differentiation of productive services in Kunming. This method helps determine spatial dependencies and the degree of spatial correlation between regions and their surroundings.

While spatial regression models, such as the Spatial Lag Model (SLM) and Spatial Durbin Model (SDM), are useful for modeling the relationships between variables across space, they are more focused on explaining global spatial dependencies and often overlook local spatial patterns. In contrast, Local Moran’s I is better suited for identifying specific regions with high or low concentrations of productive services, and for detecting spatial heterogeneity that may be missed by global models.

Anselin proposed the local index of spatial autocorrelation, LISA, which is the most common index reflecting spatial autocorrelation [31,32]. Among them, the LISA is the decomposition form of the global Moreland index, which can be used to further measure the degree of spatial correlation between the region and the surrounding region, and its expression is as follows:


$$I_{i}=\frac{\left(x_{i}-\overset{―}{x}\right)}{S^{2}}\sum_{j}w_{ij}(x_{j}-\overset{―}{x})$$ (6)

Anselin Local Moran’s I, combined with a statistical Z-test at a 95% confidence level, can identify spatially correlated patterns such as HH (high-high clustering), LL (low-low clustering), LH (high-value outliers next to low values), and HL (low-value outliers next to high values).

Moran’s I typically ranges from −1–1. A value greater than 0 indicates positive autocorrelationand that less than 0 indicates negative autocorrelation. Generally, positive autocorrelation is more common than negative autocorrelation. When Moran’s I approaches 0, it indicates a random spatial distribution with no significant spatial autocorrelation.

#### 2.3.4. Geodetector.

Geodetector is an innovative spatial statistical method used to detect spatial differentiation of geographic elements and identify their driving factors [33]. Compared to traditional geospatial statistical techniques, Geodetector has distinct advantages: it does not assume linear relationships, is not affected by multicollinearity, and allows for flexible variable types and spatial scales. These strengths make it increasingly popular in urban spatial studies. Unlike traditional regression-based methods such as multiple linear regression or spatial regression models, which require assumptions of linearity, normality, and independence, Geodetector is particularly suited for analyzing complex spatial systems with non-linear relationships and potential collinearity among variables. Additionally, while spatial lag or spatial error models mainly focus on explaining spatial dependence structures, Geodetector directly quantifies the explanatory power of individual factors and their interactions, making it more effective for uncovering the driving forces behind the spatial differentiation of productive services in heterogeneous urban environments like Kunming.

The method consists of four modules: factor detection, risk detection, interaction detection, and ecological detection. In this study, the factor detection module is employed to identify statistically significant independent variables and assess their explanatory power in relation to the dependent variables. The interaction detection module is employed to assess potential interactions between independent variables and determine their direction and type.

(1) Factor detection

The factor detection module evaluates the explanatory power of each influencing factor X on the target factor Y, measured by the q-value. The expression is as follows:


$$q=1-\frac{\sum_{h=1}^{n}N_{h}\sigma_{h}^{2}}{N\sigma_{h}^{2}}=1-\frac{SSW}{SST}$$ (7)

$$SSW=\sum_{h=1}^{n}N_{h}\sigma_{h}^{2},SST=N\sigma_{h}^{2}$$ (8)

Where h is the stratification of factor Y or factor X; $N_{h}$ and N are the number of units in layer h and the whole area, respectively. $\sigma^{2}$ and $\sigma_{h}^{2}$ are the variances of the Y values of the layer h and the whole region, respectively.

(2) **Interaction Detection**

The interaction detector is mainly used to compare the sum of q values of two factors $\left(q_{1}+q_{2}\right)$ with the q value obtained after superposition of two factor layers, and detect whether there is interaction between risk factors.

**Fig 2:**
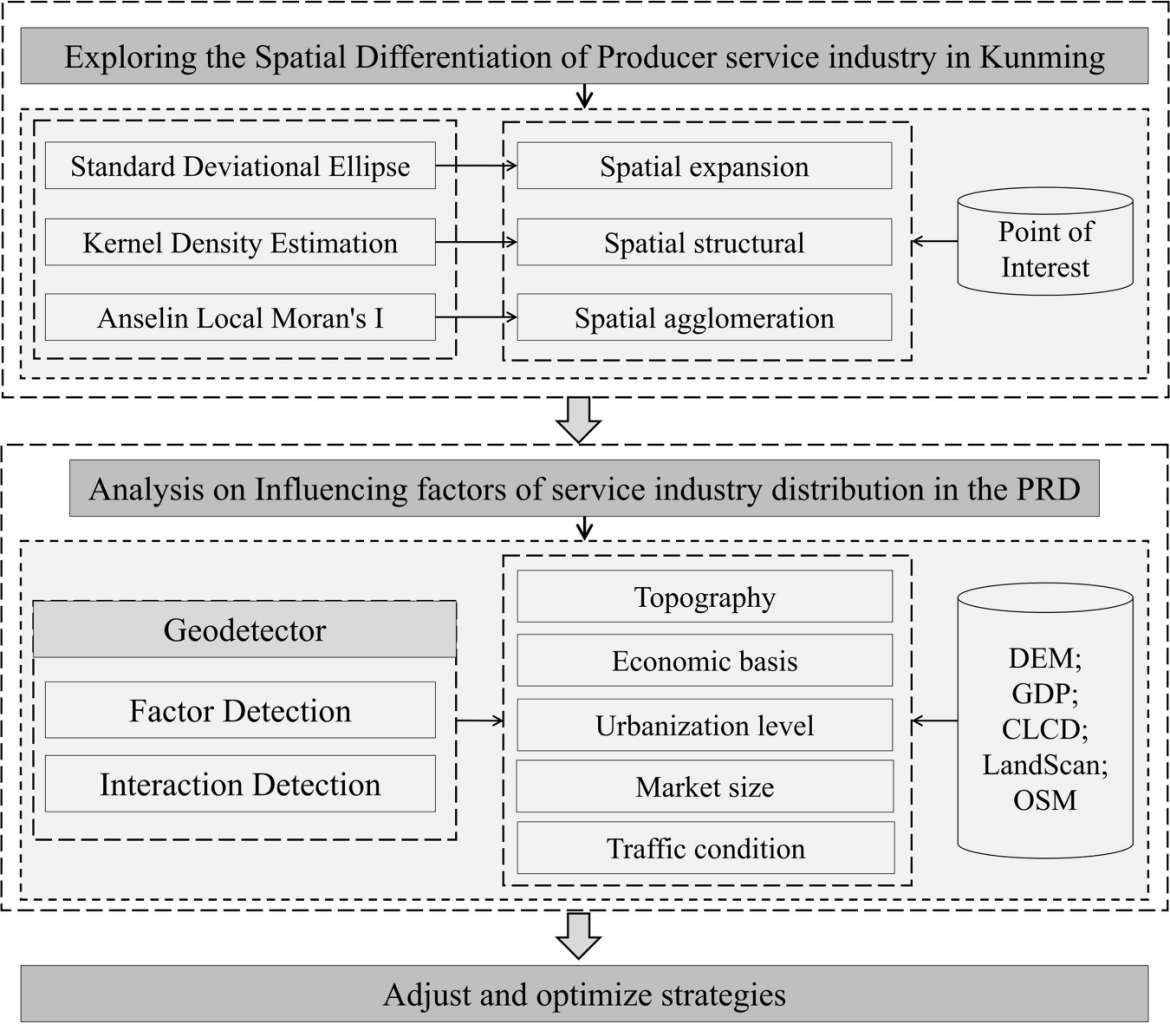
Research framework.

Building upon the classification of productive services, this study utilizes SDE, KDE, and Anselin’s Local Moran’s I to investigate the spatial differentiation of productive services. Additionally, geographic detectors are employed to quantitatively analyze the factors influencing the spatial differentiation of productive services in Kunming. This approach aims to better understand the issues within the internal structure of productive services and propose strategic recommendations. The specific research framework is presented in Fig 2.

## 3. Results

### 3.1. Spatial differentiation and evolution of productive services in Kunming

#### 3.1.1. Spatial layout analysis based on SDE.

(1) SDE of the whole productive services (WPS)

**Fig 3:**
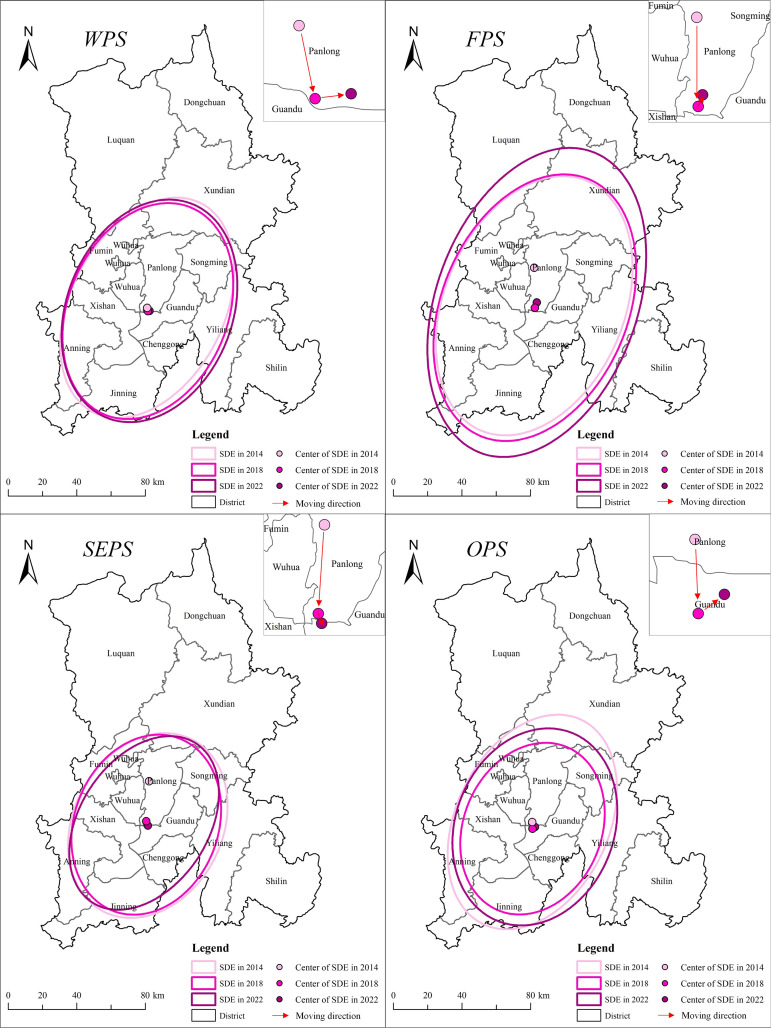
Orientation distribution of productive services in Kunming. Note: Republished from [http://bzdt.ch.mnr.gov.cn/] under a CC BY license, with permission from [the Ministry of Natural Resources of the People’s Republic of China], original copyright [2022].

As shown in Fig 3, the distribution pattern of WPS enterprises in Kunming during 2014–2022 generally presents a “south (slightly west) -north (slightly east)” spatial pattern with the central urban area as the core, and its spatial dynamic evolution characteristics are stable, with the overall trend shifting toward the southeast (Fig 3). As can be seen from Table 3, from the perspective of distribution center, the center of gravity of distribution is located in the Panlong region. The coordinates of the center of gravity point first shifted southeast from 102.752E and 25.059N in 2014 to 102.756E and 25.041N in 2018, and then shifted northeast to 102.765E and 25.042N in 2022. The center of gravity of distribution has experienced the change process of “northwest - southeast – northeast”, and the whole shows a trend of southeast migration. The reason for the shift of focus may lie in: Kunming actively responds to the national “One Belt, One Road” development strategy, as a radiation center facing South Asia and Southeast Asia, its development focus shifted to the south is conducive to better play this strategic positioning. At the same time, under the urban planning, Kunming city mainly expanded to the southeast after 2000, especially the establishment of Chenggong New District drove the rapid development of Kunming city, making the distribution center of producer service enterprises in Kunming shift southwards as a whole.

**Table 3:** SDE analysis of productive services in Kunming in 2014, 2018, and 2022.

Types	Years	Center of gravity		Major axis/km	Minor axis/km	Oblateness	Area/km²	Rotation Angle/°
		Longitude	Latitude					
WPS	2014	102.752	25.059	67.88	41.46	0.39	8841.91	27.46
	2018	102.756	25.041	64.78	43.16	0.33	8782.33	22.66
	2022	102.765	25.042	66.46	44.77	0.33	9348.38	21.65
FPS	2014	102.762	25.269	78.32	48.57	0.38	11950.45	23.25
	2018	102.766	25.059	80.48	50.21	0.38	12696.69	23.04
	2022	102.776	25.087	92.74	54.25	0.42	15804.65	20.38
SEPS	2014	102.762	25.269	54.83	34.71	0.37	5980.13	33.35
	2018	102.748	25.059	53.33	38.88	0.27	6515.15	21.04
	2022	102.756	25.036	55.86	40.25	0.28	7062.41	27.71
OPS	2014	102.751	25.055	65.42	41.27	0.37	8481.81	25.75
	2018	102.753	25.020	51.14	37.21	0.27	5977.49	22.92
	2022	102.765	25.029	58.38	42.82	0.27	7854.07	21.10

The long axis of the SDE of WPS in Kunming first decreased from 67.88 km in 2014 to 64.78 km in 2018, and then increased to 66.46 km in 2022, indicating that the distribution of productive services in the north-south direction remained stable. The short axis of the SDE first increased from 41.46 km in 2014 to 43.16 km in 2018, and then continued to increase to 44.77 km in 2022, indicating that the distribution of WPS was expanding from northwest to southeast. The flattening of the standard ellipse first decreased from 0.39 in 2014 to 0.33 in 2018, and then remained stable until 2022, indicating that the directionality of the spatial distribution of WPS experienced a trend of intensification – weakening – stability over time.

The SDE area first decreased from 8841.91 km² in 2014 to 8782.33 km² in 2018, and then increased to 9348.38 km² in 2022, indicating that the distribution of WPS in Kunming experienced a process of first shrinking and then expanding during 2014–2022. The rotation Angle was first rotated from 27.46° in 2014 to 22.66° in 2018 and then to 21.65° in 2022, indicating that the overall performance of WPS in Kunming was expanding to the southeast.

(2) SDE of different types of productive services

The distribution pattern of FPS, SEPS, and OPS enterprises showed a “south (slightly west) -north (slightly east)” distribution pattern with central urban areas as the core, and the distribution center of gravity showed a southward migration trend.

The distribution center of FPS is located in Panlong District, and its distribution center moves to the southeast. The long axis of the SDE continued to increase from 78.32 km in 2014 to 92.74 64.78 km in 2022, and the short axis continued to increase from 48.57 km in 2014 to 54.25 km in 2022, indicating that the FPS continued to spread to the periphery. The flatness of the standard ellipse first increased from 0.38 in 2014 to 0.42 in 2022, indicating that its spatial distribution is increasingly directional. The SDE area continued to increase from 11950.45 km² in 2014 to 15804.65 km² in 2022, indicating that the distribution range of FPS in Kunming continued to expand during this period.

The distribution center of SEPS moved southward from Panlong District to Guandu District. The long axis of the SDE decreased from 54.83 km in 2014 to 53.33 km in 2018, and then increased to 55.86 km in 2022, with fluctuations but overall stability during the period. The short axis continues to increase from 34.71 km in 2014 to 40.25 km in 2022, indicating that the distribution of SEPS in the northwest to southeast is expanding. The oblateness of the standard ellipse decreases from 0.37 in 2014 to 0.28 in 2022, indicating that the directionality of its spatial distribution has weakened. The SDE area continued to increase from 5980.13 km² in 2014 to 7062.41 km² in 2022, indicating that the distribution range of SEPS in Kunming continued to expand during this period.

The distribution center of OPS migrated from Panlong District to Guandu District, and the migration direction showed a process of first southeast and then northeast. The long axis of the SDE decreases from 65.42 km in 2014 to 51.14 km in 2018 and then increases to 58.38 km in 2022, while the short axis decreases from 41.27 km in 2014 to 37.21 km in 2018 and then increases to 42.82 km in 2022. It shows that OPS have experienced the distribution trend of first polarization and then diffusion. The flatness of the standard ellipse decreases from 0.37 in 2014 to 0.27 in 2022, indicating that the directionality of its spatial distribution has weakened. The SDE area decreased from 8481.81 km² in 2014 to 5977.49 km² in 2018, and then increased to 7854.07 km² in 2022, indicating that the distribution of OPS in Kunming experienced a trend of first contraction and then expansion during this period.

#### 3.1.2. Spatial structure analysis based on KDE.

**Fig 4:**
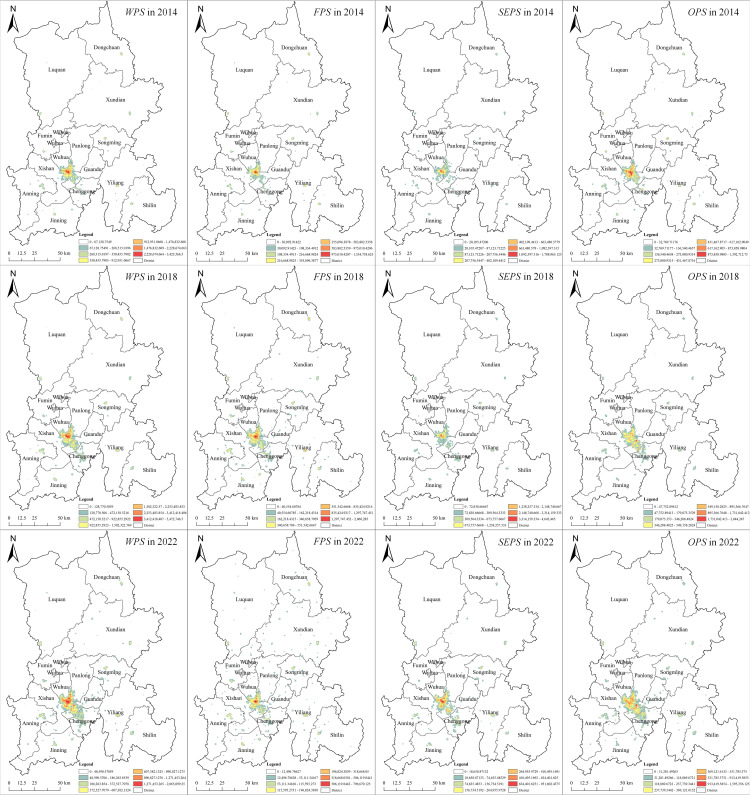
KED analysis of productive services in Kunming. Note: Republished from [http://bzdt.ch.mnr.gov.cn/] under a CC BY license, with permission from [the Ministry of Natural Resources of the People’s Republic of China], original copyright [2022].

The POI data of WPS were imported into ArcGIS, appropriate distance thresholds and pixel sizes were selected, and the kernel density values were re-graded by natural breakpoint classification method. Finally, the overall and various types of WPS in 2014, 2018 and 2022 were obtained (Fig 4).

First, the distribution range of the kernel density of WPS spread significantly to the southeast from 2014 to 2018, and the overall range was relatively stable from 2018 to 2022, but the distribution density increased significantly, which may be related to the policy process of developing WPS in Kunming City. During the “13th Five-Year Plan” period, the Kunming municipal government began to prioritize productive services and accelerated the establishment of related enterprises. In the “14th Five-Year Plan” period, productive services experienced significant development towards specialization and high-end services. Related enterprises further consolidated and expanded, leading to the formation of a spatial pattern that has largely developed in a static manner.

Second, the overall spatial structure of productive services in Kunming has gradually expanded from a single-center structure in 2014 to a multi-center structure in 2022. Among them, SEPS and OPS also show a relatively obvious multi-center distribution. This may be related to the industrial development and personnel training of Chenggong New Area. By shifting from traditional agriculture to modern service industry and high-tech industry, Chenggong New Area develops key industries such as Internet of Things, cloud computing, big data center and artificial intelligence, and promotes the development of productive services; Through the construction of university town, it actively introduces and trains talents, and provides talents support for productive services. The FPS maintains a single center structure with the central city as the core, which may be related to the stage of economic development. The FPS of Kunming are mainly concentrated in the old urban areas such as Wuhua District, Panlong District, Guandu District and Xishan District. As the core areas of Kunming’s service economy development, these areas have relatively mature financial service facilities and institutions. Although a financial industrial park is planned in Chenggong New Area, compared with the old city, its development time is shorter, and the agglomeration and expansion of FPS need time to be gradually realized.

Second, the overall spatial structure of productive services in Kunming has gradually expanded from a single-center structure in 2014 to a multi-center structure in 2022. Among them, SEPS and OPS also show a relatively obvious multi-center distribution. This may be related to the industrial development and personnel training of Chenggong New Area. By shifting from traditional agriculture to modern service industry and high-tech industry, Chenggong New Area develops key industries such as Internet of Things, cloud computing, big data center and artificial intelligence, and promotes the development of productive services; Through the construction of university town, it actively introduces and trains talents, and provides talents support for productive services. The FPS maintains a single center structure with the central city as the core, which may be related to the stage of economic development. The FPS of Kunming are mainly concentrated in the old urban areas such as Wuhua District, Panlong District, Guandu District and Xishan District. As the core areas of Kunming’s service economy development, these areas have relatively mature financial service facilities and institutions. Although a financial industrial park is planned in Chenggong New Area, compared with the old city, its development time is shorter, and the agglomeration and expansion of FPS need time to be gradually realized.

#### 3.1.3. Spatial agglomeration analysis based on spatial autocorrelation.

According to the agglomeration effect of the Anselin Local Moran’s I, productive services in Kunming can be divided into five types of spatial agglomeration: (1) H-H clusters, indicating that the region and adjacent regions have a high level of enterprise distribution density; (2) H-L clusters, indicating that the region has a high level of enterprise distribution density, and the adjacent region has a low enterprise distribution density; (3) L-H clusters, indicating that the distribution density of enterprises in the region is low, and the distribution density of enterprises in the adjacent region is high; (4) L-L clusters, which means that the region and its adjacent regions have a low level of enterprise distribution density; (5) Insignificant clusters.

**Fig 5:**
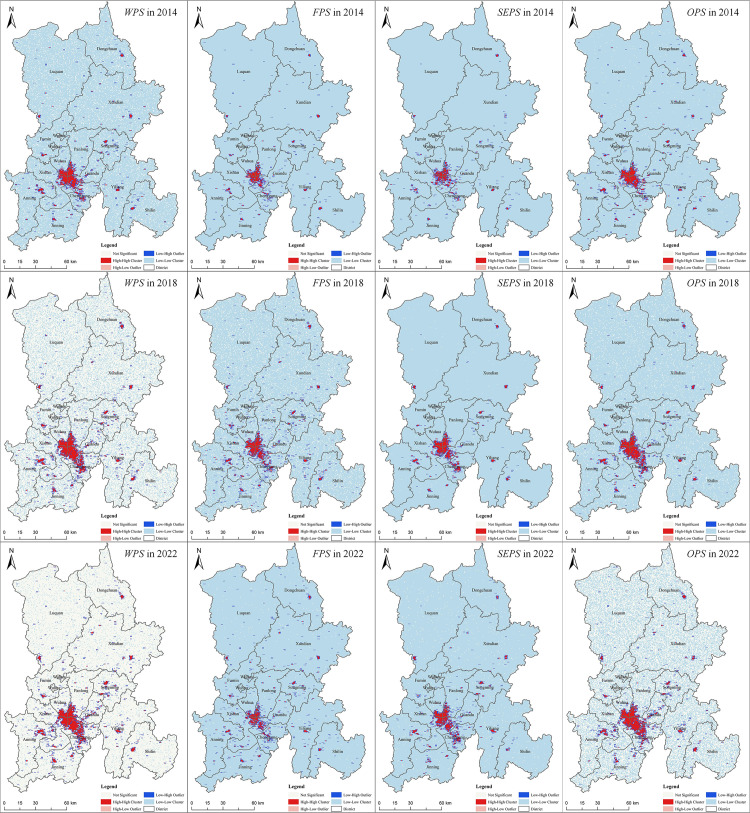
LISA diagram of productive services in Kunming. Note: Republished from [http://bzdt.ch.mnr.gov.cn/] under a CC BY license, with permission from [the Ministry of Natural Resources of the People’s Republic of China], original copyright [2022].

From the overall productive services and its various types of LISA diagram (Fig 5) during 2014–2022, in general, H-H clusters and L-L clusters are dominant. As can be seen from the LISA diagram of the overall productive services, in 2014, its H-H clusters were concentrated in the main urban area, and showed a star-dot distribution in other core areas of districts and counties. These areas have long been the center of social and economic activities in Kunming. Due to the intensive population, industrial and economic activities and policy support, the spatial agglomeration of producer service enterprises is significant. L-L clusters are distributed in most areas outside the main urban areas. These regions are relatively sparse in population and economic activity and lack the basis for the development of productive services. By 2018, H-H clusters increased, while L-L clusters were gradually scattered. This is because under the guidance of the “14th Five-Year Plan” on the development of productive services, related enterprises continue to expand in the core areas of the city, and begin to emerge in the suburbs of new agglomeration areas. By 2022, there will be a decrease in the number of high cluster areas in the periphery, which means that the regions with higher development level of productive services will have a certain degree of dispersion in spatial distribution, which may be related to the high-end specialization of productive services and the transition of industrial energy levels. However, the L-L clusters are gradually transformed into an insignificant area, which means that the difference in the development level of productive services between regions is narrowing, which may be related to policy intervention, infrastructure improvement and other factors.

According to the LISA diagram of various types of productive services, the L-L clusters of FPS and SEPS are relatively more continuous, while the H-H clusters trend of OPS is more pronounced. In the LISA diagram for FPS, in 2014, H-H clusters were concentrated in the main urban area and the core urban areas of surrounding districts and counties, while L-L clusters were continuously and completely distributed outside the main urban area. By 2018, the H-H clusters had significantly expanded, while the L-L clusters gradually fragmented due to the emergence of non-significant areas. By 2022, the H-H clusters in the central urban area had shrunk, while the L-L clusters had regained a more complete layout. The LISA diagram for SEPS shows that the H-H clusters underwent a process of expansion followed by fragmentation, while the L-L clusters exhibited little change overall, with a slight fragmentation trend in recent years. In the LISA diagram for OPS, the H-H clusters cover a wide area and show a continuous expansion trend. The L-L clusters, on the other hand, displayed a continuous decentralization pattern.

### 3.2. Analysis of influencing factors of spatial differentiation of productive services in Kunming

#### 3.2.1. Construction of probe factor system.

Productive services provide intermediate input services for industrial production processes, as well as for the primary and tertiary industries. As a key factor in accelerating the integration of the secondary and tertiary industries, there are numerous influencing factors affecting their spatial layout and agglomeration. Based on a review of the literature and relevant theoretical research [34,35], and considering the specific context of the study area, this paper primarily selects factors such as terrain, economic foundation, urbanization level, market size, and traffic conditions to analyze their impact on the spatial distribution of productive service enterprises in Kunming (Table 4). The dependent variable is the number of productive service enterprises in each grid.

**Table 4:** The detection indicator system for influencing factors.

Detection Indicator	Remark	Data type
Topography(X_1_)	Average slope per grid	ASTER GDEM 30M resolution digital elevation data
Economic basis(X_2_)	Total GDP per grid	China GDP spatial distribution kilometer grid dataset
Urbanization level(X_3_)	Area of built-up area per grid	The 30 m annual land cover datasets and its dynamics in China(CLCD)
Market size(X_4_)	Number of resident population per grid	LandScan Global
Traffic condition(X_5_)	Road length per grid	OpenStreetMap(OSM)

(1) Terrain: Different terrain will have an impact on the spatial expansion of the city, and will also affect the layout of urban industrial space through the price of land class rent. In this study, DEM was used to extract the surface slope as the terrain influence variable.
(2) Economic foundation: Regions with better economic foundation have natural advantages, such as better transportation conditions, good business environment and abundant labor resources, which are more attractive to producer service enterprises to settle in. This study uses per capita GDP to represent the urban economic base.
(3) Urbanization level: Urbanization level can reflect the development stage and basis of a city, and urban built-up area is an important aspect to reflect urbanization level. In this study, CLCD was used to extract urban impervious area to represent urbanization level.
(4) Market size: Generally speaking, producer service enterprises located in large population size are more likely to obtain labor resources. This study uses the number of permanent residents to reflect the market size.
(5) Transportation conditions: Good transportation infrastructure can promote the agglomeration and development of the service industry, improve the accessibility and coverage of the service industry, weaken the spatial barriers to the flow of production factors, and promote the optimal allocation of factors such as manpower, capital and technology in a wider range. In this study, OSM data is used to calculate the road network density, and then reflect the urban traffic conditions.

#### 3.2.2. Factor detection results.

To identify the core driving factors of the spatial differentiation of productive services in Kunming, factor detection was performed for each influencing factor. The results indicate that the P-value for each factor is less than 0.01, suggesting a statistically significant impact on the spatial differentiation of productive services in Kunming. Thus, the factor detection results are considered reliable.

The explanatory power of each influencing factor is further assessed. The value of q reflects the explanatory power of each independent variable with respect to the dependent variable, ranging from 0 to 1. The larger the q value is, the stronger its explanatory ability is; conversely, the weaker it is. As shown in Table 5, the q-values for all types of productive services generally align with those of the overall pattern. Among all factors influencing productive services, traffic conditions (X_5_) have the highest explanatory power, followed by economic foundation (X_2_), indicating that these are the core driving factors for the spatial differentiation of productive services in Kunming. The improvement of transportation conditions is conducive to strengthening intra-regional and inter-regional economic ties, promoting economic agglomeration, reducing transaction costs, and expanding the market scope of productive services. At the same time, areas with a better economic foundation tend to have a large market scale and frequent economic activities, which are more conducive to the agglomeration and development of productive services.

**Table 5:** Factor detection results of various types of productive services.

q-value	X_1_	X_2_	X_3_	X_4_	X_5_
Y	0.797726	0.907568	0.437485	0.883108	0.981716
Y_1_	0.766239	0.834825	0.329719	0.896497	0.981948
Y_2_	0.773540	0.926384	0.312090	0.870043	0.994501
Y_3_	0.823404	0.915121	0.380672	0.845653	0.952757

The meanings of X_1_ to X_5_ are shown in Table 4.

From the dimensions of the influence of various factors on different types of productive services, transportation conditions (X_5_) have the strongest explanatory power for all types of productive services; secondly, economic base (X_2_) has a stronger explanatory power for the layout of SEPS and OPS; and market size has a stronger explanatory power for the layout of FPS. FPS is a typical industry with increasing returns to scale, and large-scale unified market is conducive to its professional development. At the same time, due to the externalization and market-oriented service demand of FPS, the expansion of market scale will directly affect its external demand and market transaction activity, so it has a strong explanatory power for the layout of FPS.

#### 3.2.3. Interactive detection Results.

This study analyzes the explanatory power of relevant influencing factors on the spatial distribution of productive services in Kunming by using the interaction detection of geographical detector. The interactive detection results of overall productive services and various types of productive services are shown in Table 6.

**Table 6:** Interactive detection results of various types of productive services.

Y	X_1_	X_2_	X_3_	X_4_	X_5_
X_1_	0.797726				
X_2_	0.994246	0.907568			
X_3_	0.995240	0.995122	0.437485		
X_4_	0.999323	0.999961	0.993597	0.883108	
X_5_	0.998039	0.998153	0.985984	0.998047	0.981716
Y_1_	X_1_	X_2_	X_3_	X_4_	X_5_
X_1_	0.766239				
X_2_	0.985185	0.834825			
X_3_	0.988366	0.98289	0.329719		
X_4_	0.998213	0.999669	0.985164	0.896497	
X_5_	0.996985	0.994443	0.987457	0.994405	0.981948
Y_2_	X_1_	X_2_	X_3_	X_4_	X_5_
X_1_	0.773540				
X_2_	0.995683	0.926384			
X_3_	0.995183	0.996619	0.31209		
X_4_	0.999138	0.999979	0.995299	0.870043	
X_5_	0.999830	0.998419	0.996686	0.998971	0.994501
Y_3_	X_1_	X_2_	X_3_	X_4_	X_5_
X_1_	0.823404				
X_2_	0.995708	0.915121			
X_3_	0.995717	0.995566	0.380672		
X_4_	0.999295	0.999960	0.990617	0.845653	
X_5_	0.994699	0.998209	0.961919	0.996952	0.952757

(1) The detection results of the interaction of overall productive services and all types are nonlinear or double-factor enhanced, indicating that the impact of interaction on the spatial distribution of service industry is greater than that of single factor.
(2) The interaction between economic base and market size ranks first, which further indicates the dependence of productive services on the consumer market and the promotion of employment.
(3) After the interaction, the explanatory power of factors with smaller q-values is greatly improved.
(4) The interaction test results for productive services indicate that neither the overall distribution nor the individual factors exhibit nonlinear weakening or independence. This suggests that the spatial distribution of productive services in Kunming is not driven by a single factor, but rather by the combined effects of various influencing factors.

## 4. Discussion

The development of productive services has important strategic significance for promoting economic restructuring, promoting deep industrial integration, improving urban economic efficiency and enabling high-quality economic development [36]. Since it contains very rich industry types, a classified study on it is helpful to understand the spatial distribution characteristics and influencing factors of different types of productive services, and provide a basis for the government to formulate more accurate and effective policies.

Based on the analysis of the spatial distribution characteristics of productive services in Kunming, it can be seen that, first, the distribution center of the whole and all types of productive services in Kunming showed a trend of southwards shift, and the distribution scope expanded to the southeast, while the spatial distribution direction of the whole, SEPS and OPS weakened, indicating that under the guidance of the national strategy and development orientation, Productive services in Kunming have expanded rapidly and spread to surrounding areas, promoting the industrial upgrading and transformation of productive services within the city. Alonso’s urban land use model emphasizes that transportation costs are an important factor affecting land use [37]. In Kunming, with the improvement of transportation infrastructure, especially the relocation of the municipal government to Chenggong District, the transportation infrastructure in the southern and southeastern areas has been perfected, reducing the transportation costs in these areas and enhancing their attractiveness to productive services. This further promoted the expansion of productive service industries to these regions. Moreover, the spatial distribution of FPS has become increasingly directional, indicating that the agglomeration effect of Kunming in the financial industry is being strengthened, and the construction of its regional international financial service center and Kunming International Financial Town attracts all kinds of financial institutions to develop in Kunming. In comparison, the Chengdu-Chongqing Economic Circle exhibits a more pronounced spatial concentration of productive services. Chengdu, in particular, has developed a multi-center structure for its FPS sector, with significant financial hubs emerging in areas like the Chengdu Tianfu New Area. This decentralization is supported by targeted policies and infrastructure investments aimed at promoting balanced regional development [38,39]. Second, the spatial structure of the overall productive services, SEPS and OPS in Kunming has developed from a single center to a multi-center structure. This model helps alleviate the excessive concentration pressure of a single center and promotes balanced regional development. And the FPS have maintained a single center spatial structure, indicating that they have always maintained their dependence on the urban center, which may be because financial services need to be close to the decision-making center, information center and customer concentration area. Urban centers generally have better transport connections and infrastructure, which is conducive to the operation and growth of financial institutions. In contrast, Chengdu has developed a multi-center structure for its FPS sector, with significant financial hubs emerging in areas like the Chengdu Tianfu New Area. This decentralization is supported by targeted policies and infrastructure investments aimed at promoting balanced regional development.

From the analysis of influencing factors of productive services in Kunming, it can be seen that transportation conditions and economic foundation dominate the development of productive services in Kunming. In addition, market scale has a great impact on the distribution of FPS. As the link of intra-regional and inter-regional economic relations, transportation infrastructure has significant externality and network, and its perfection will affect the agglomeration effect of productive services [40]. Regions with a strong economic foundation can provide more resources and market opportunities, attract producer service enterprises to gather, and promote the professional and diversified development of industries. The expansion of market size can provide more business opportunities and demand, promote the specialization and diversification of productive services, and thus improve economic efficiency [41]. At present, the development of productive services in Kunming is relatively lagging behind, and there are still a series of problems such as agglomeration effect is not prominent and scale effect is not obvious. Comparatively, Chengdu and Chongqing have leveraged their economic scale and infrastructure to foster a more robust FPS sector. Chengdu, for instance, has established itself as a financial hub with a diverse range of financial services, supported by policies that encourage innovation and attract talent. In the context of industrial structure adjustment, policy support, infrastructure development, and an open market environment for cooperation, Kunming, as a key node of the “One Belt, One Road” initiative, should comprehensively enhance productive services through various measures. These include improving transportation infrastructure, expanding market size, promoting the professionalization and high-end development of productive services, and accelerating the establishment of industrial integration and agglomeration areas.

Building upon the classification of productive services, this study analyzes the spatial differentiation of productive services in Kunming using socio-economic and POI data. It also investigates the factors influencing this differentiation, to provide recommendations for the future development of productive services. Regarding the research content, current studies on the spatial characteristics of productive services primarily focus on the spatial coordination between productive services and manufacturing industries [42], and rarely discuss its classification. According to the latest classification standard of productive services industry, this study classifies its types, which is conducive to obtaining more accurate and targeted research results. In terms of research methods and data, the current research mainly uses statistical yearbook data, economic census data, etc. to study the spatial layout and evolution of productive services by using various indexes and GIS-based spatial analysis methods based on municipal, district, and county divisions [43,44]. The spatial characteristics and influencing factors are rarely discussed from more detailed time scale and more diversified geospatial data. Based on the 1 km grid scale, this study uses POI data, SDE, KDE, and Anselin Local Moran’s I to explore the spatial distribution characteristics of productive services, and uses DEM, CLCD, OSM, socio-economic data, and geographic detector to explore its influencing factors, and obtains more detailed and targeted research results. It makes up for the lack of spatial dimension consideration in the existing research. In terms of research areas, existing studies pay more attention to the empirical study of productive services in economically developed cities, but pay less attention to large cities with important geographical significance in less developed areas. Based on the particularity of node cities along the “One Belt, One Road”, this study analyzes the spatial differentiation and influencing factors of productive services in Kunming, which is conducive to enriching the empirical research on productive services.

Despite the valuable insights gained, this research has several limitations. First, as previously discussed, the Geodetector model effectively identifies spatial associations but does not capture causal relationships, which are crucial for understanding the mechanisms driving spatial differentiation. Future studies should consider employing more advanced spatial econometric models to address this gap and provide deeper insights. Second, the third-party datasets utilized in this study offer valuable insights into Kunming’s productive service sector. However, potential biases in these datasets may influence the accuracy of the findings. Future research should address these biases through multi-source data validation and enhanced quality control measures. Lastly, this study did not fully explore the temporal dimension of the influencing factors. Future research should examine how the spatial distribution of productive services evolves over time to offer more robust insights into the future development of Kunming and the central Yunnan city cluster. Additionally, the current study lacks a systematic temporal analysis of trend evolution. Future research could incorporate time-series clustering or spatiotemporal autocorrelation methods to more systematically track the evolution of spatial differentiation trends, thereby enhancing the depth and breadth of the study.

## 5. Conclusions

The optimization and enhancement of productive services are essential prerequisites for the development of the economy. Building upon the classification of productive services, this study utilizes POI data to examine their spatial differentiation characteristics and employs multi-source data to quantitatively assess the factors influencing these services. This approach aims to identify the overall and specific spatial layout characteristics, as well as the influencing factors of productive services in Kunming, and subsequently propose recommendations for the adjustment and optimization of productive services in the city.

The results show that at present, the productive services in Kunming generally presents the characteristics of southward migration and diffusion, and the traffic conditions and economic basis are the main influencing factors. From the perspective of each subdivision type, SEPS and OPS spread significantly, and developed from single-center structure to multi-center structure, and the traffic conditions and economic basis had the most significant impact on it. On the other hand, the FPS cluster and develop in a single-center structure with the central city as the core, and the traffic conditions and market scale have the most significant influence on it. In the post-epidemic era under the requirements of high-quality development of the country, the overall development of Kunming’s productive services has rebounded and improved, but there are still problems such as fragmented development of various types of industries and insufficient collaborative service ability of industries.

To address these challenges and promote the high-quality development of Kunming’s productive services, we propose the following policy recommendations:

(1) Enhance Infrastructure Connectivity: Prioritize the expansion of transportation networks, including rail and road links, and accelerate the development of digital infrastructure such as 5G networks and data centers to support the digital transformation of productive services.
(2) Implement Targeted Industrial Policies: Develop detailed industrial plans for productive services, leveraging Kunming’s unique resources and industrial base. Provide financial, tax, and policy support to enterprises, particularly small and medium-sized ones, to encourage growth. Establish specialized industrial parks and improve supporting facilities to attract enterprises and foster industrial clusters.
(3) Promote Technological Innovation: Increase investment in technological innovation by supporting enterprises in technological upgrades, product development, and business model innovations. Strengthen cooperation between industry, academia, and research institutions to facilitate the commercialization of technological achievements. Establish public innovation platforms and enhance intellectual property protection to stimulate innovation.
